# Curiosity driven reinforcement learning for motion planning on humanoids

**DOI:** 10.3389/fnbot.2013.00025

**Published:** 2014-01-06

**Authors:** Mikhail Frank, Jürgen Leitner, Marijn Stollenga, Alexander Förster, Jürgen Schmidhuber

**Affiliations:** ^1^Dalle Molle Institute for Artificial IntelligenceLugano, Switzerland; ^2^Facoltà di Scienze Informatiche, Università della Svizzera ItalianaLugano, Switzerland; ^3^Dipartimento Tecnologie Innovative, Scuola Universitaria Professionale della Svizzera ItalianaManno, Switzerland

**Keywords:** artificial curiosity, intrinsic motivation, reinforcement learning, humanoid, iCub, embodied AI

## Abstract

Most previous work on *artificial curiosity* (AC) and *intrinsic motivation* focuses on basic concepts and theory. Experimental results are generally limited to toy scenarios, such as navigation in a simulated maze, or control of a simple mechanical system with one or two degrees of freedom. To study AC in a more realistic setting, we *embody* a curious agent in the complex iCub humanoid robot. Our novel reinforcement learning (RL) framework consists of a state-of-the-art, low-level, reactive control layer, which controls the iCub while respecting constraints, and a high-level curious agent, which explores the iCub's state-action space through information gain maximization, learning a world model from experience, controlling the actual iCub hardware in real-time. To the best of our knowledge, this is the first ever embodied, curious agent for real-time motion planning on a humanoid. We demonstrate that it can learn compact Markov models to represent large regions of the iCub's configuration space, and that the iCub explores *intelligently*, showing *interest* in its physical constraints as well as in objects it finds in its environment.

## 1. Introduction

Reinforcement Learning (RL) (Barto et al., 1983; Sutton and Barto, 1998; Kaelbling et al., 1996) allows an *agent* in an *environment* to learn a *policy* to maximize some sort of *reward*. Rather than optimizing the policy directly, many RL algorithms instead learn a value function, defined as expected future discounted cumulative reward. Much of early RL research focused on discrete states and actions instead of continuous ones dealt with by function approximation and feature-based representations.

An RL agents needs to *explore* its environment. Undirected exploration methods (Barto et al., 1983), rely on randomly selected actions, and do not differentiate between already explored regions and others. Contrastingly, directed exploration methods can focus the agent's efforts on novel regions. They include the classic and often effective *optimistic initialization*, go-to the least-visited state, and go-to the least recently visited state.

### 1.1. Artificial curiosity (AC)

Artificial Curiosity (AC) refers to directed exploration driven by a world model-dependent value function designed to direct the agent toward regions where it can learn something. The first implementation (Schmidhuber, 1991b) was based on an *intrinsic reward* inversely proportional to the predictability of the environment. A subsequent AC paper (Schmidhuber, 1991a) emphasized that the reward should actually be based on the *learning progress*, as the previous agent was motivated to fixate on inherently unpredictable regions of the environment. Subsequently, a probabilistic AC version (Storck et al., 1995) used the well known Kullback-Leibler (KL) divergence (Lindley, 1956; Fedorov, 1972) to define non-stationary, intrinsic rewards reflecting the changes of a probabilistic model of the environment after new experiences. Itti and Baldi (2005) called this measure *Bayesian Surprise* and demonstrated experimentally that it explains certain patterns of human visual attention better than previous approaches.

Over the past decade, robot-oriented applications of curiosity research have emerged in the closely related fields of Autonomous Mental Development (AMD) (Weng et al., 2001) and Developmental Robotics (Lungarella et al., 2003). Inspired by child psychology studies of Piaget (Piaget and Cook, 1952), they seek to learn a strong base of useful skills, which might be combined to solve some externally posed task, or built upon to learn more complex skills.

Curiosity-driven RL for developmental learning (Schmidhuber, 2006) encourages the learning of appropriate skills. Skill learning can be made more explicit by identifying learned skills (Barto et al., 2004) within the option framework (Sutton et al., 1999). A very general skill learning setting is assumed by the PowerPlay framework, where skills actually correspond to arbitrary computational problem solvers (Schmidhuber, 2013; Srivastava et al., 2013).

Luciw et al. (2011) built a curious planner with a high-dimensional sensory space. It learns to perceive its world and predict the consequences of its actions, and continually plans ahead with its imperfect but optimistic model. Mugan and Kuipers developed QLAP (Mugan and Kuipers, 2012) to build predictive models on a low-level visuomotor space. Curiosity-Driven Modular Incremental Slow Feature Analysis (Kompella et al., 2012) provides an intrinsic reward for an agent's progress toward learning new spatiotemporal abstractions of its high-dimensional raw pixel input streams. Learned abstractions become option-specific feature sets that enable skill learning.

### 1.2. Developmental robotics

Developmental Robotics (Lungarella et al., 2003) seeks to enable robots to learn to do things in a general and adaptive way, by trial-and-error, and it is thus closely related to AMD and the work on curiosity-driven RL, described in the previous section. However, developmental robotic implementations have been few.

What was possibly the first AC-like implementation to run on hardware (Huang and Weng, 2002) rotated the head of the SAIL robot back and forth. The agent/controller was rewarded based on reconstruction error between its improving internal perceptual model and its high-dimensional sensory input.

AC based on learning progress was first applied to a physical system to explore a playroom using a Sony AIBO robotic dog. The system (Oudeyer et al., 2007) selects from a variety of pre-built behaviors, rather than performing any kind of low-level control. It also relies on a remarkably high degree of random action selection, 30%, and only optimizes the immediate (next-step) expected reward, instead of the more general delayed reward.

Model-based RL with curiosity-driven exploration has been implemented on a Katana manipulator (Ngo et al., 2012), such that the agent learns to build a tower, without explicitly rewarding any kind of stacking. The implementation does use pre-programmed *pick and place* motion primitives, as well as a set of specialized pre-designed features on the images from an overhead camera.

A curiosity-driven modular reinforcement learner has recently been applied to surface classification (Pape et al., 2012), using a robotic finger equipped with an advanced tactile sensor on the fingertip. The system was able to differentiate distinct tactile events, while simultaneously learning behaviors (how to move the finger to cause different kinds of physical interactions between the sensor and the surface) to generate the events.

The so-called hierarchical curiosity loops architecture (Gordon and Ahissar, 2011) has recently enabled a 1-DOF LEGO Mindstorms arm to learn simple reaching (Gordon and Ahissar, 2012).

Curiosity implementations in developmental robotics have sometimes used high dimensional sensory spaces, but each one, in its own way, greatly simplified the action spaces of the robots by using pre-programmed high-level motion primitives, discretizing motor control commands, or just using very, very simple robots. We are unaware of any AC (or other intrinsic motivation) implementation, which is capable of learning in, and taking advantage of a complex robot's high-dimensional configuration space.

Some methods learn internal models, such as hand-eye motor maps (Nori et al., 2007), inverse kinematic mappings (D'Souza et al., 2001), and operational space control laws (Peters and Schaal, 2008), but these are not curiosity-driven. Moreover, they lack the generality and robustness of full-blown path planning algorithms (Latombe et al., 1996; LaValle, 1998; Li and Shie, 2007; Perez et al., 2011).

### 1.3. The path planning problem

The *Path Planning Problem* is to find motions that pursue goals while deliberately avoiding arbitrary non-linear constraints, usually obstacles. The ability to solve the path planning problem in practice is absolutely critical to the eventual goal of deploying complex/humanoid robots in unstructured environments. The recent textbook, “Planning Algorithms” (LaValle, 2006), offers many interesting approaches to planning motions for complex manipulators. These are expensive algorithms, which search the configuration space to generate trajectories that often require post-processing. Thus robots, controlled by algorithmic planners, are typically very deliberate and slow, first “thinking,” often for quite some time, then executing a motion, which would be simple and intuitive for humans.

### 1.4. Reactive control

In the 1980s, a control strategy emerged, which was completely different from the established *plan first, act later* paradigm. The idea was to use potential fields (Khatib, 1986; Kim and Khosla, 1992), and/or dynamical systems (Schoner and Dose, 1992; Iossifidis and Schoner, 2004, 2006), and/or the sensor signals directly (Brooks, 1991) to generate control commands fast, without searching the configuration space. Control is based on some kind of local gradient, which is evaluated at the robot's current configuration. As a result, sensors and actuators are tightly coupled in a fast, light weight action/observation loop, allowing a robot to react quickly and smoothly to changing circumstances. Nevertheless, reactive controllers are shortsighted and prone to getting stuck in local minima/maxima, making them relatively bad path planners.

### 1.5. A curious confluence

In this paper, we introduce a curiosity-driven reinforcement learner for the iCub humanoid robot (Metta et al., 2008), which autonomously learns a powerful, reusable solver of motion planning problems from experience controlling the actual, physical robot.

The application of RL to the path planning problem (or more precisely the process of embodying the agent at a sufficiently low level of control) has allowed us to incorporate two approaches, planning and reactive control, which for the most part have been treated separately by roboticists until now. The integrated system benefits from both approaches while avoiding their most problematic drawbacks, and we believe it to be an important step toward realizing a practical, feasible, developmental approach to real, non-trivial robotics problems. Furthermore, the system is novel in the following ways:

1. In contrast to previous implementations of artificial curiosity and/or intrinsic motivation in the context of developmental robotics, our system learns to control many degrees of freedom (DOFs) of a complex robot.
2. Planning algorithms typically generate reference trajectories, which must then be passed to a controller. Our RL system, on the other hand, learns control commands directly, while still yielding a resolution complete planner. This greatly simplifies many practical issues that arise from tracking a reference trajectory and results in a lighter, faster action/observation loop.
3. Rather than relying on reactive control to generate entire motions, we only use it to implement actions. Thus the completeness of the planner is preserved, although its robustness is improved by the added capacity of each action react to unforeseen and/or changing constraints.

## 2. Material and methods

In order to build a developmental learning system capable of exploiting the iCub's high DOF configuration space, we begin by looking at the path planning literature, where there exist two classes of algorithms, capable of generating high dimensional reference trajectories. Single query algorithms, such as Rapidly Exploring Random Trees (RRT) (LaValle, 1998; Perez et al., 2011), interpolate two points in configuration space, without reusing knowledge from one query to the next. Multiple query algorithms on the other hand, such as Probabilistic Road Maps (PRM) (Latombe et al., 1996; Sun et al., 2005), store a compressed representation of the configuration space and satisfy queries by operating on that data structure, rather than searching the high DOF configuration space directly. In the case of PRM, the configuration space is represented by a graph, which can even be grown incrementally (Li and Shie, 2007). PRM's compact, incrementally expandable representation of known motions makes it a likely antecedent to or template for a development learning system, but there are several problems, which are all related to *separation* between planning and control.

To build up a PRM planner, one must first sample the configuration space to obtain a set of vertices for the graph. The samples are then interpolated by trajectories, which form the set of edges that connect the vertices. The feasibility of each sample (vertex) and trajectory (edge) must be preemptively verified, typically by forward kinematics and collision detection computations, which collectively amount to a computationally expensive pre-processing step. The configuration of the robot *must* remain on the verified network of samples and trajectories at all times, or there may be unwanted collisions. This implies that all the trajectories in the graph must also be controllable, which is in general difficult to verify in simulation for complex robots, such as the iCub, which exhibit non-linear dynamics (due to do friction and deformation) and are thus very difficult to model faithfully. If these problems can be surmounted, then a PRM planner can be constructed, however, the configuration of the robot's workspace must be static, because moving anything therein may affect the feasibility of the graph edges.

All of these problems can be avoided by *embodying the planner* and giving the system the capacity to *react*. If there were a low-level control system, which could enforce all necessary constraints (to keep the robot safe and operational) in real time, then the planner could simply try things out, without the need to exhaustively and preemptively verify the feasibility of each potential movement. In this case, reference trajectories would become unnecessary, and the planner could simply store, recall, and issue control commands directly. Lastly, and perhaps most importantly, with the capacity to react in real time, there would be no need to require a static workspace.

This new *embodied planner* would differ from its antecedent PRM planner in several important ways. There would be no need to require that the configuration of the robot be *on* any of the graph edges. In fact the graph would no longer represent a network of distinct trajectories, but rather the *topology* of the continuous configuration space. Each edge would no longer represent a particular trajectory, but rather a more general kind of *action* that implements something like *try to go to that region of the configuration space*. Such actions would be available not when the true robot configuration is *on* a graph vertex, but rather when it is *near* that vertex. The actions may or may not succeed depending on the particular initial configuration of the robot when the action was initiated as well as the configuration of the workspace, which must not necessarily be static.

Allowing the planner to control the hardware directly offers considerable benefits, but it also requires a more complex representation of the configuration space than the *plan first, act later* paradigm did. Whereas the PRM planner made do with a simple graph, representing a network of trajectories, the embodied version seems to require a probabilistic model, which can cope with actions that may have a number of different outcomes. In light of this requirement, the embodied planner begins to look like a Markov Decision Process (MDP), and in order to exploit such a planner, the state transition probabilities, which govern the MDP, must first be learned. However, this presents a problem in that experiments (trying out actions) are very expensive when run on robotic hardware, which is bound to real time, as opposed to simulations, which can be run faster than real time, or parallelized, or both. Therefore, an efficient exploration method is absolutely critical, which motivates our use of curiosity-driven RL.

### 2.1. Action implementation

**Figure 1:**
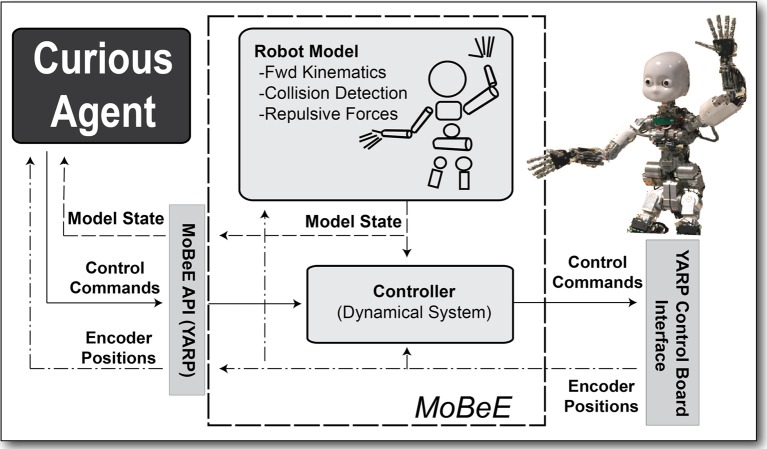
**MoBeE and the iCub**. MoBeE **(left)** prevents the iCub humanoid robot **(right)** from colliding with the table. Semi-transparent geometries represent force fields, and when these collide with one another (shown in red), they generate repulsive, constraint forces, which in this case push the hands away from the table surface.

**Figure 2:**
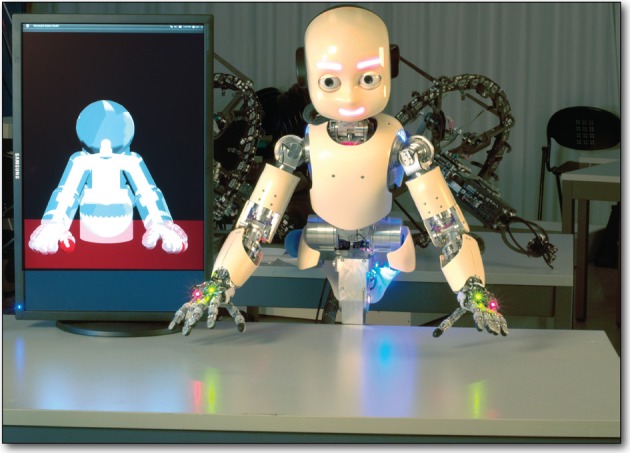
**The modular behavioral environment (MoBeE) architecture**. MoBeE implements low-level control and enforces all necessary constraints to keep the robot safe and operational in real time, such that the curious RL agent **(left)** is able to experiment with arbitrary control commands. A kinematic/geometric model of the iCub humanoid robot **(top)** is driven by streaming motor encoder positions from the hardware **(right)**. The model computes fictitious constraint forces, which repel the robot from collisions, joint limits, and other infeasibilities. These forces, *f*_*i*_(*t*) in Equation (1), are passed to the controller **(middle)**, which computes the attractor dynamics that governs the actual movement of the robot.

We have put considerable energy into developing the low-level control system described above, the Modular Behavioral Environment (MoBeE; Figures 1 and 2) (Frank et al., 2012), the details of which are beyond the scope of this paper. In this section, we describe MoBeE only insofar as to define the notion of *action* as it pertains to our RL system.

MoBeE controls the robot constantly, at a high frequency, according to the following second order dynamical system:

(1) $$\text{M}\ddot{q}(t)+\text{C}\dot{q}(t)+\text{K}(q(t)-q^{*})=\sum f_{i}(t)$$

The vector function *q*(*t*) ∈ ℝ^*n*^ is the robot configuration, and the matrices **M, C**, and **K** contain mass, damping, and spring constants, respectively. The position vector q^*^ is an attractor, and constraints on the system are implemented by forcing it via *f*_*i*_(*t*), which provides automatic avoidance of kinematic infeasibilites having to do with joint limits, cable lengths, and collisions.

An action, for the purposes of RL, means setting the attractor q^*^ to some desired configuration. When such an action is taken, *q*(*t*) begins to move toward q^*^. The action terminates either when the dynamical system settles or when a timeout occurs. The action may or may not settle on q^*^, depending on what constraint forces, *f*_*i*_(*t*) are encountered during the transient response.

### 2.2. State-action space

**Figure 3:**
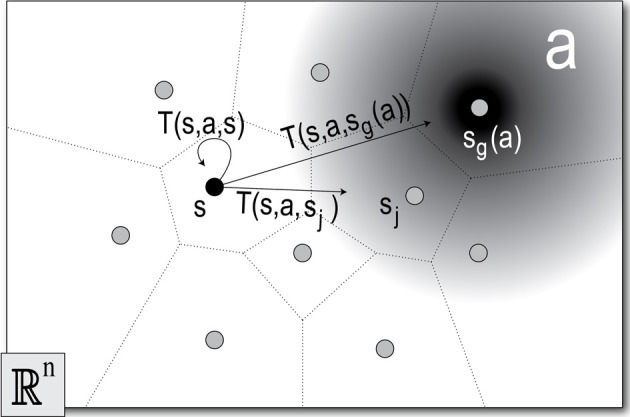
**The discrete state-action space**. The sample set *Q* = {*q*_*j*_|*j* = 1 … *m*} (dots) defines the Voronoi regions, or states *S* = {*s*_*j*_|*j* = 1 … *m*} (bounded by dotted lines). An action *a* (gradient), exploits MoBeE's attractor dynamics to pull the robot toward some goal state, *s*_*g*_(*a*) ∈ *S*. When the robot is in the initial state, *q*(*t*_0_) ∈ *s*, and the agent selects *a*, MoBeE switches on the attractor (Equation 1) at the point *q*_*g*_ ∈ *s*_*g*_(*a*). The agent then waits for the dynamical system to settle or for a timeout to occur, and at some time, *t*_1_, checks which of the states, *s*_*j*_ contains the final real valued configuration of the robot, *q*(*t*_1_). Often the state-action, (*s, a*), terminates in the goal state *s*_*g*_(*a*), but sometimes, due to constraint forces, it does not. This gives rise to a set of state transition probabilities *T*(*s, a*) = {*T*(*s, a, s*′_1_), *T*(*s, a, s*′_2_), …, *T*(*s, a, s*′_*m*_)}, which correspond to the states, {*s*_*j*_|*j* = 1 … *m*}.

The true configuration of the robot at any time *t* can be any real valued *q* ∈ ℝ^*n*^, however, in order to define a tractable RL problem, we discretize the configuration space (Figure 3) by selecting *m* samples, *Q* = {*q*_*j*_|*j* = 1 … *m*} ⊂ ℝ^*n*^. The sample set *Q* defines a set of states^1^ *S* = {*s*_*j*_|*j* = 1 … *m*}, such that $\overset{m}{\underset{j\;=\;1}{\cup }}s_{j}={\mathbb{R}}^{n}$. Each state, *s*_*j*_ ∈ *S*, is the Voronoi region associated with the corresponding sample, *q*_*j*_ ∈ *Q*. That is to say, each sample, *q*_*j*_ ∈ ℝ^*n*^, defines a state, *s*_*j*_ ⊂ ℝ^*n*^, where every point, *q* ∈ *s*_*j*_, is closer^2^ to *q*_*j*_ than to any other point *q* ∈ *Q*. The states in our Markov model are the sets, *s* ∈ *S*, not the points, *q* ∈ *Q*, and to say that the robot is in some particular state, *s*, at some particular time, *t*, means that the real valued configuration of the robot, *q*(*t*) ∈ *s*.

An action is defined by setting MoBeE's attractor, q^*^ = *q*_*g*_ (Equation 1), where *q*_*g*_ ∈ *Q* is the sample in some goal state *s*_*g*_(*a*). When an action is tried, the robot moves according to the transient response, *q*(*t*), of the dynamical system, which eventually settles at *q*(*t* → ∞) = *q*_∞_. However, depending on the constraint forces encountered, it may be that *q*_∞_ ∈ *s*_*g*_(*a*) or not.

#### 2.2.1. Connecting states with actions

An action, *a, intends* to move the robot to some goal state *s*_*g*_(*a*), a waypoint along the path that will eventually be generated by the reinforcement learner. But which states should be connected to which other states? In order that our Markov model develops into an effective path planner, we want to connect each state to its *k* nearest neighbors^3^ in a way that makes sense with respect to the dimensionality of the configuration space, *n*. To this end, we choose *k* = 2^*n*^, as an *n*-dimensional hypercube has 2^*n*^ vertices.

With each state, *s*, is associated a set of actions, *A*(*s*), which intend to move the robot from *s* to each of *k* nearby goal states, *A*(*s*) = {*a*_*g*_|*g* = 1 … *k*}, and the set of all possible actions, *A*, can therefore be expressed as the union of the action sets belonging to each state, $A=\overset{m}{\underset{s\;=\;1}{\cup }}A(s)$.

This notion of connecting neighboring states makes intuitive sense given the problem domain at hand and the resulting Markov model resembles the Roadmap graph used by the PRM planner (Latombe et al., 1996). Although the action set, *A*, is quite large (|*A*| = |*S*|), each state only has access to the actions, *A*(*s*), which lead to its *k* nearest neighbors (|*A*(*s*)| = *k*). Therefore, the number of state-actions remains linear in the number of states. We advise the reader that wherever the standard state-action notation, (*s, a*), is used, it is implied that *a* ∈ *A*(*s*).

#### 2.2.2. Modeling transition probabilities

Although each action *intends* to move the robot to some particular goal state, in principal they can terminate in *any* state in the set {*s*_*j*_|*j* = 1 … *m*}. Therefore, we must learn state transition probabilities to represent the connectivity of the configuration space. A straightforward way of doing this would be to define a probability distribution over all possible outcomes *s*_*j*_ for each state-action (*s, a*):

(2) $$T(q_{\infty }\in s_{j}|s,a)=\left\{\begin{array}{c} p(q_{\infty }\in s_{1}|s,a)\\ p(q_{\infty }\in s_{2}|s,a)\\ \vdots \\ p(q_{\infty }\in s_{m}|s,a) \end{array}\right\}$$

To build up the distributions, *T*(*q*_∞_ ∈ *s*_*j*_|*s, a*), we would simply initialize all probabilities to zero and then count the occurrences of observed transitions to the various states, *s*_*j*_, resulting from the various state-actions (*s, a*). We would, however, find this approach to be relatively wasteful, because much of the state-action space is deterministic. In practice, we find that there are only three kinds of distributions that come out of applying RL algorithms to our Markov model. A state-action, (*s, a*), can terminate deterministically in the goal state *s*_*g*_(*a*) (Equation 3), it can terminate deterministically in some other state *s*_*j*_ ≠ *s*_*g*_(*a*) (Equation 4), or it can be truly non-deterministic (Equation 5), although the non-zero components of *T* are always relatively few compared to the number of states in the model.

(3) $$p(q_{\infty }\in s_{j}|s,a)=\{\begin{array}{ll} 1 & \text{if }s_{j}=s_{g}(a)\\ 0 & \text{if }s_{j}\neq s_{g}(a) \end{array}$$

(4) $$p(q_{\infty }\in s_{j}|s,a)=\{\begin{array}{ll} 1 & \text{if }s_{j}=s^{*}\neq s_{g}(a)\\ 0 & \text{if }s_{j}\neq s^{*} \end{array}$$

(5) $$p(q_{\infty }\in s_{j}|s,a)\;\{\begin{array}{l} >0\text{ if }s_{j}\in S_{1}\\ =0\text{ if }s_{j}\in S_{0} \end{array}|S_{0}\cup S_{1}=S,|S_{0}|\gg |S_{1}|$$

This is intuitive upon reflection. Much of the configuration space is not affected by constraints, and actions always complete as planned. Sometimes constraints are encountered, such as joint limits and cable length infeasibilities, which deflect the trajectory in a predictable manner. Only when the agent encounters changing constraints, typically non-static objects in the robot's operational space, do we see a variety of outcomes for a particular state-action. However, even in this case, the possible outcomes, *s*′, are a relatively small number of states, which are usually in the neighborhood of the initial state, *s*. We have never constructed an experiment, using this framework, in which a particular state-action, (*s, a*), yields more than a handful of possible outcome states, *s*′.

We can and have used distributions of the form shown in Equation (2) to model the outcomes of state-actions in our RL framework. However, we have found a better way to represent the distribution, which is more parsimonious, and facilitates a better AC signal.

### 2.3. Artificial curiosity

What is interesting? For us humans, *interestingness* seems closely related to the rate of our learning progress (Schmidhuber, 2006). If we try doing something, and we rapidly get better at doing it, we are often interested. Contrastingly, if we find a task trivially easy, or impossibly difficult, we do not enjoy a high rate of learning progress, and are often bored. We model this phenomenon using the information theoretic notion of *information gain*, or KL divergence.

#### 2.3.1. KL divergence

KL Divergence, *D*_*KL*_ is defined as follows, where *P*_*j*_ and *T*_*j*_ are the scalar components of the discrete probability distributions *P* and *T*, respectively.

(6) $$D_{KL}(P||T)=\sum_{j}\ln\left(\frac{P_{j}}{T_{j}}\right)P_{j}$$

For our purposes, *T* represents the estimated state transition probability distribution (Equation 2) for a particular state-action, (*s, a*), after the agent has accumulated some amount of experience. Once the agent tries (*s, a*) again, an *s*′ is observed, and the state transition probability distribution for (*s, a*) is updated. This new distribution, *P*, is a better estimate of the state transition probabilities for (*s, a*), as it is based on more data.

By computing *D*_*KL*_(*P*||*T*), we can measure how much our Markov model improved by trying the state-action, (*s, a*), and we can use this *information gain* to reward our curious agent. Thus, the agent is motivated to improve its model of the state-action space, and it will gravitate toward regions thereof, where learning is progressing quickly.

There is, however, a problem. The KL divergence is not defined if there exist components of *P* or *T*, which are equal to zero. This is somewhat inconvenient in light of the fact that for our application, most of the components of most of the distributions, *T* (Equation 2), *are* actually zero. We must therefore initialize *P* and *T* cleverly.

Perhaps the most obvious solution would be to initialize *T* with a uniform distribution, before trying some action for the first time. After observing the outcome of the selected action, *P* would be defined and *D*_*KL*_(*P*||*T*) computed, yielding the *interestingness* of the action taken.

Some examples of this kind of initialization are given in Equations (7–10)^4^. Clearly the approach solves the numerical problem with the zeros, but it means that initially, *every* action the agent tries will be equally interesting. Moreover, *how* interesting those first actions are, |*D*_*KL*_(*P*||*T*)|, depends on the size of the state space.

(7) $$D_{KL}(\{1,2,1\}||\{1,1,1\})=0.0589$$

(8) $$D_{KL}(\{2,1,1\}||\{1,1,1\})=0.0589$$

(9) $$D_{KL}(\{1,1,2,1,1\}||\{1,1,1,1,1\})=0.0487$$

(10) $$D_{KL}(\{1,1,1,2,1,1,1\}||\{1,1,1,1,1,1,1\})=0.0398$$

The first two examples, Equations (7), (8), show that regardless of the outcome, all actions generate the same numerical interestingness the first time they are tried. While not a problem in theory, in practice this means our robot will need many tries to gather enough information to differentiate the boring, deterministic states from the interesting, non-deterministic ones. Since our actions are designed to take the agent to a goal state, *s*_*g*_(*a*), it would be intuitive if observing a transition to *s*_*g*_(*a*) were less interesting than observing one to some other state. This would drastically speed up the learning process.

The second two examples, Equations (9), (10) show that the *interestingness* of that first try decreases in larger state spaces, or alternatively, small state spaces are numerically more *interesting* than large ones. This is not a problem if there is only one learner operating in a single state-action space. However, in the case of a multi-agent system, say one learner per body part, it would be convenient if the intrinsic rewards gotten by the different agents were numerically comparable to one another, regardless of the relative sizes of those learners' state-action spaces.

In summary, we have two potential problems with KL Divergence as a reward signal:

1. Slowness of initial learning
2. Sensitivity to the cardinality of the distributions

Nevertheless, in many ways, KL Divergence captures exactly what we would like our curious agent to focus on. It turns out we can address both of these problems by representing *T* with an array of variable size, and initializing the distribution optimistically with respect to the expected behavior of the action (*s, a*).

#### 2.3.2. Dynamic state transition distributions

By compressing the distributions *T* and *P*, i.e., not explicitly representing any bins that contain a zero, we can compute the KL divergence between only their non-zero components. The process begins with *T* and *P* having no bins at all. However, they grow in cardinality as follows: Every time we observe a novel *s*′ as the result of trying a state-action (*s, a*), we append a new bin to the distribution *T*(*s, a*), and initialize it with a 1, and copy it to yield *P*(*s, a*). Then, since we just observed (*s, a*) result in *s*′, we increment the corresponding bin in *P*(*s, a*), and compute *KL*(*P*||*T*). This process is formalized in Algorithm 1.

**Algorithm 1:**
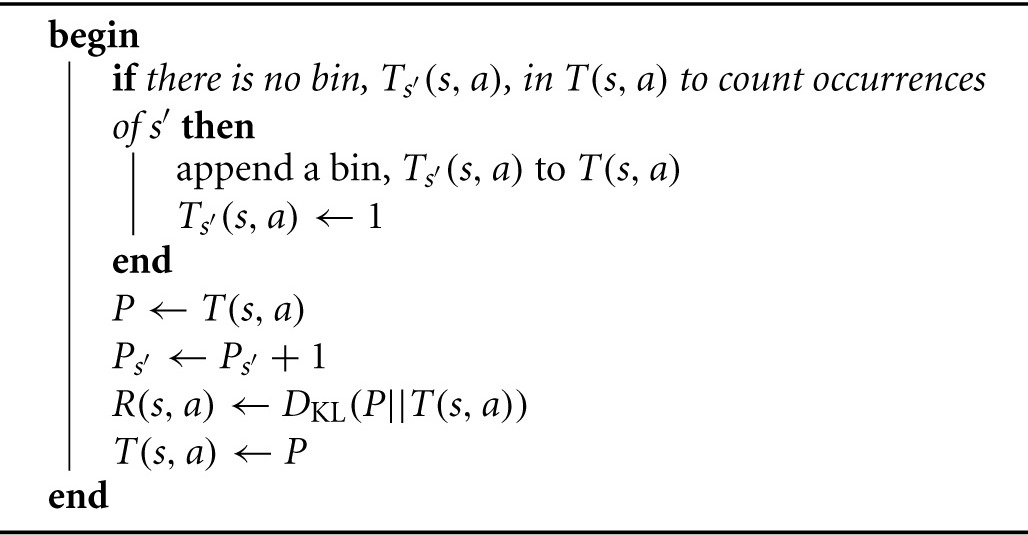
Observe(*s,a,s′,T(s, a),R(s, a))*

**Algorithm 2:**
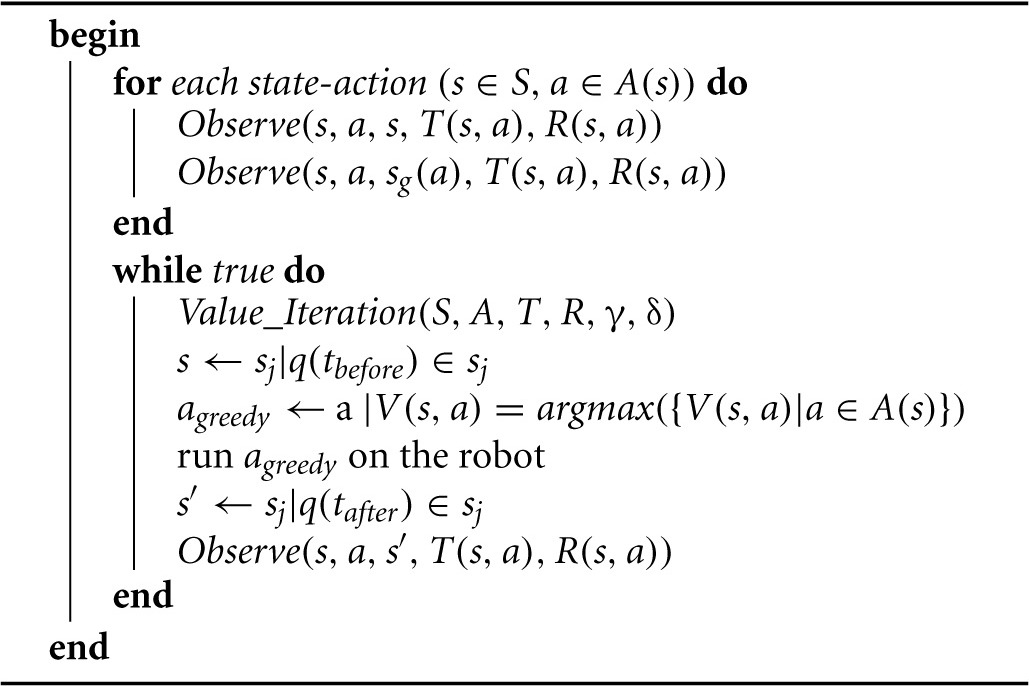
Curious_Explore(S,A,T,R,γ,δ)

The optimistic initialization is straightforward. Initially, the distribution *T*(*s, a*) is empty. Then we *observe* (Algorithm 1) that (*s, a*) fails, leaving the agent in the initial state, *s*. The KL divergence between the trivial distributions {1} and {2} is 0, and therefore, so is the reward, *R*(*s, a*). Next, we *observe* that (*s, a*) succeeds, moving the agent to the intended goal state, *s*_*g*_(*a*). The distribution, *T*(*s, a*), becomes non-trivial, a non-zero KL divergence is computed, and thus *R*(*s, a*) gets an optimistically initialized reward, which does not depend on the size of the state-action space. Algorithm 2 describes the steps of this optimistic initialization, and Table 1 shows how *T*(*s, a*) and *R*(*s, a*) develop throughout the initialization process.

**Table 1:** **Initialization of state transition probabilities**.

**Observation**	***T***	***P***	***R = D_KL_(P||T)***
–	{}	{}	–
*s*_*i*_	{1}	{2}	0
*s*_*g*_(*a*)	{2,1}	{2,2}	0.0589

The distributions *T*, as initialized above, are compact and parsimonious, and they faithfully represent the most likely outcomes of the actions. Moreover, the second initialization step yields a non-zero KL Divergence, which is not sensitive to the size of the state space. Importantly, the fact that our initialization of the state transition probabilities provides an initial measure of *interestingness* for each state-action allows us, *without choosing parameters*, to optimistically initialize the reward matrix with well defined *intrinsic rewards*. Consequently, we can employ a greedy policy, and aggressively explore the state-action space while focusing extra attention on the most *interesting* regions. As the curious agent explores, the intrinsic rewards decay in a logical way. A state-action, which deterministically leads to its goal state (Table 2) is less interesting over time than a state-action that leads to some other state (Table 3), and of course most *interesting* are state-actions with more possible outcomes (Table 4).

**Table 2:** **A predictable action ends in the predicted state**.

**Observation**	***T***	***P***	***R = D_KL_(P||T)***
init	{2,1}	{2,2}	0.0589
*s*_*g*_(*a*)	{2,2}	{2,3}	0.0201
*s*_*g*_(*a*)	{2,3}	{2,4}	0.0095
*s*_*g*_(*a*)	{2,4}	{2,5}	0.0052

**Table 3:** **A predictable action ends in a surprising state**.

**Observation**	***T***	***P***	***R = D_KL_(P||T)***
init	{2,1}	{2,2}	0.0589
*s*_*j*_	{2,2,1}	{2,2,2}	0.0487
*s*_*j*_	{2,2,2}	{2,2,3}	0.0196
*s*_*j*_	{2,2,3}	{2,2,4}	0.0103

**Table 4:** **An unpredictable action**.

**Observation**	***T***	***P***	***R = D_KL_(P||T)***
init	{2,1}	{2,2}	0.0589
*s*_*a*_	{2,2,1}	{2,2,2}	0.0487
*s*_*b*_	{2,2,2,1}	{2,2,2,2}	0.0345
*s*_*c*_	{2,2,2,2,1}	{2,2,2,2,2}	0.0283
*s*_*g*_(*a*)	{2,2,2,2,2}	{2,3,2,2,2}	0.0142
*s*_*a*_	{2,3,2,2,2}	{2,3,3,2,2}	0.0133
*s*_*b*_	{2,3,3,2,2}	{2,3,3,3,2}	0.0125

### 2.4. Reinforcement learning

At the beginning of section 2, we made the claim that a PRM planner's compact, incrementally expandable representation of known motions makes it a likely antecedent to a developmental learning system. Furthermore, we observed that many of the weaknesses of PRMs can be avoided by *embodying the planner* and coupling it to a low-level reactive controller. Proxied by this low-level controller, the planner is empowered to try out arbitrary control signals, however, it does not necessarily know what will happen. Therefore, the PRM's original model of the robot's state-action space, a simple graph, is insufficient, and a more powerful, probabilistic model, an MDP is required. Thus, modeling the robot-workspace system using an MDP arises naturally from the effort to improve the robustness of a PRM planner, and accordingly, Model-Based RL is the most appropriate class of learning algorithms to operate on the MDP.

Having specified what *action* means in terms of robot control (section 2.1), described the layout and meaning of the state-action space (section 2.2), and defined the way in which intrinsic reward is computed according to the AC principal (section 2.3), we are ready to incorporate these pieces in a Model-Based RL system, which develop into a path planner as follows: Initially, sets of states and actions will be chosen, according to some heuristic(s), such that the robot's configuration space is reasonably well covered and the RL computations are tractable. Then, the state transition probabilities will be learned for each state-action pair, as the agent explores the MDP by moving the robot about. This exploration for the purposes of model learning will be guided entirely by the intrinsic reward defined in section 2.3, and the curious agent will continually improve its model of the iCub and its configuration space. In order to exploit the planner, an external reward must be introduced, which can either be added to or replace the intrinsic reward function.

The MDP, which constitutes the path planner, is a tuple, <*S, A, T, R*, γ>, where *S* is a finite set of *m* states, *A* is a finite set of actions, *T* is a set of state transition probability distributions, *R* is a reward function, and γ is a discount factor, which represents the importance of future rewards. This MDP is somewhat unusual in that not all of the actions *a* ∈ *A* are available in every state *s* ∈ *S*. Therefore, we define sets, *A*(*s*), which comprise the actions *a* ∈ *A* that are available to the agent when it finds itself in state *s*, and $A=\overset{m}{\underset{s\;=\;1}{\cup }}A(s)$. The set of state transition probabilities becomes $T:\overset{m}{\underset{s\;=\;1}{\cup }}A(s)\times S\to [0,1]$, and in general, the reward function becomes $R:\overset{m}{\underset{s\;=\;1}{\cup }}A(s)\times S\to \mathbb{R}$, although the intrinsic reward, $R_{\text{intrinsic}}:\overset{m}{\underset{s\;=\;1}{\cup }}A(s)\to \mathbb{R}$, varies only with state-action pairs (*s, a*), as opposed to state-action-state triples (*s, a, s*′). The state transition probabilities, *T*, are learned by curious exploration (Algorithm 2, γ = 0.9, δ = 0.001), the RL algorithm employed is value iteration (Algorithm 3), and the intrinsic reward is computed as shown in Algorithm 1.

**Algorithm 3:**
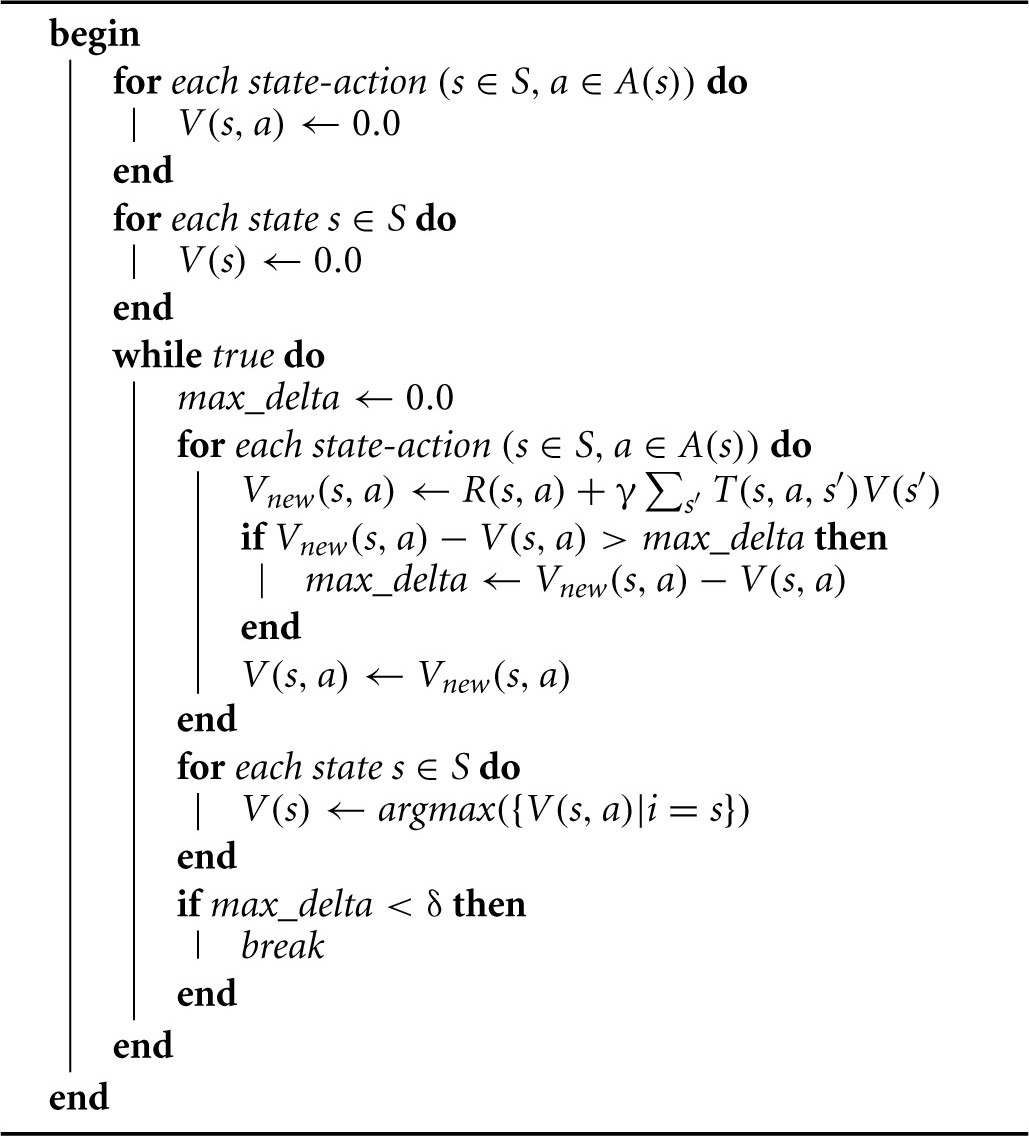
Value_Iteration(S,A,T,R,γ,δ)

## 3. Results

Here we present the results of two online learning experiments. The first one learns a motion planner for a single limb, the iCub's arm, operating in an unobstructed workspace, while other body parts remain motionless. The planner must contend with self-collisions, and infeasibilities due to the relative lengths of the cables, which move the shoulder joints. These constraints are static, in that they represent properties of the robot itself, which do not change regardless of the configuration of the workspace. Due to the static environment, a PRM planner would in principal be applicable, and the experiment provides a context in which to compare and contrast the PRM versus MDP planners. Still, the primary question addressed by this first experiment is: “To what extent does AC help the agent learn the state transition probabilities for the MDP planner in this real-world setting?”

In the second experiment, the iCub is positioned at a work table, which constitutes a large obstacle in its workspace. Three curious agents, unaware of one another's states, learn planners for the iCub's torso and two arms, respectively. One could in principal define a single curious MDP planner for the whole body, but this would result in an explosion of the state-action space such that running actual experiments on the iCub hardware would be prohibitively time consuming. The modular, parallel, multi-agent configuration of this second experiment is designed to address the question: “Can curious MDP planners scale to intelligently control the entire iCub robot?” And in observing the behavior emergent from the interactions between the 3 learners, this will be the question of primary importance. Also noteworthy, however, is that from the perspective of the arms, which do not know that the torso is moving, the table seems to be non-static. By analyzing the arm learning while disregarding the torso, one can gain insight into how the curious MDP planner copes with non-static environments, which would render the PRM planner inoperable.

### 3.1. Planning in a static environment—learning to avoid self-collisions and cable length infeasibilities

In the first experiment, “Planning in a static environment,” we compare the exploration of our artificially curious agent (AC), to two other agents using benchmark exploration strategies from the RL literature. One explores randomly (RAND), and the other always selects the state-action least tried (LT)^5^.

The state space is defined by choosing samples, which vary in 4 dimensions corresponding to three shoulder joints and the elbow. Each of these joints is sampled at 25%, 50%, and 75% of its range of motion, resulting in a 4D hyper-lattice with 81 vertices, which are connected to their 2^4^ = 16 nearest neighbors as per section 2.2.1, yielding 81 × 16 = 1296 state-actions. The intuition behind this choice of state space it comprises a compact yet reasonably well dispersed set of pre-reach poses.

The task is to find the infeasible region(s) of the configuration space, and learn the according state transition probabilities such that the agent can plan motions effectively. The task is relatively simple, but it is none the less a crucial aspect of any path planning that should take place on the iCub. Without deliberately avoiding self-collisions and cable length infeasibilities, a controller can and will break the iCub's cables, rendering it inoperable.

**Figure 4:**
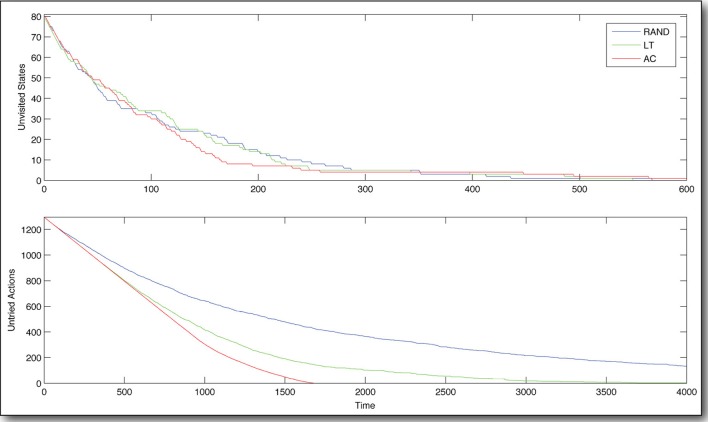
**State-action space coverage during early learning**. The policy based on Artificial Curiosity (AC) explores the state-action space most efficiently, compared to policies based on random exploration (RAND) and always selecting the least tried state-action (LT). Time is measured in state transitions.

In comparing the AC agent with the RAND agent and the LT agent, we find that AC produces, by far, the best explorer (Figure 4). In the early stages of learning, AC and LT try *only* novel actions, whereas RAND tries some actions repeatedly. Early on (before the agent has experienced about 220 state transitions), the only difference evident between AC and LT is that AC visits novel states more aggressively. This is intuitive upon reflection, as AC values states with *many* untried state-actions, and will traverse the state space to go find them, whereas LT has no global knowledge and just chooses the locally least tried state-action, regardless of where it leads. As learning continues, this key difference between AC and LT also begins to manifest in terms of the coverage of the action space. In fact, AC tries all possible state-actions in about $\frac{1}{2}$ the time it takes LT.

**Figure 5:**
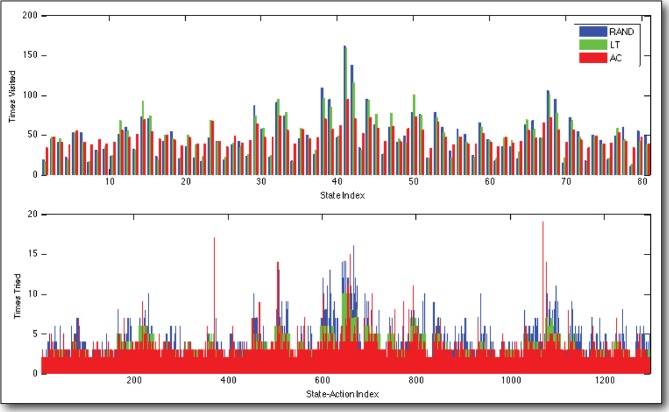
**Distributions of visits over states and tries over actions**. Our curious agent (AC) visits states and tries actions in a more uniformly than to policies based on random exploration (RAND) and always selecting the least tried state-action (LT). Note the few state-actions, which have been tried many times by AC. These are affected by the cable length constraints in the iCub's shoulder. They terminate in an unexpected way, which is *interesting* or *surprising* to the agent, and they therefore receive more attention. These data are compiled over 4000 state transitions, observed while controlling the real, physical iCub humanoid robot.

Moving on to the tabulated number of times that each state was visited and each state-action was tried, after 4000 state transitions, again we see that AC exhibits preferable behavior to LT and RAND (Figure 5). AC results in distributions of visits over states and tries over state-actions, which are more uniform than those resultant of RAND and LT. Moreover, we see a number of large spikes, where the AC agent became very interested in certain state-actions. In fact, these are the actions that run the robot into its constraints, and therefore do not cause the anticipated state transition (Equation 4). While most of the state-actions' rewards decay according to Table 2, these spikes were generated by state-actions whose rewards are governed by Table 3, and they are thus more *interesting* to the agent.

**Figure 6:**
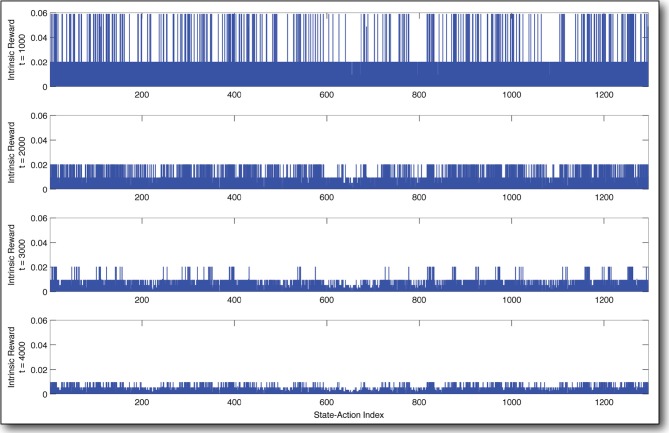
**Decay of intrinsic reward over time**. These snapshots of the reward distribution state-actions (x-axis) over time (from **top** to **bottom**) show how our curious agent becomes *bored* as it builds a better and better model of the state-action space. Time is measured in state transitions.

The decay of the intrinsic reward over the state-action space over time is shown in Figure 6. The uniformity of the decay is intuitive, since whenever there exists a spike in the reward function, the AC agent goes and gets it, thereby gaining experience and decrementing the reward for the state-action tried. Thus, differing rates of decay (Tables 1–4) govern the frequency with which the agent tries the different state-actions.

**Figure 7:**
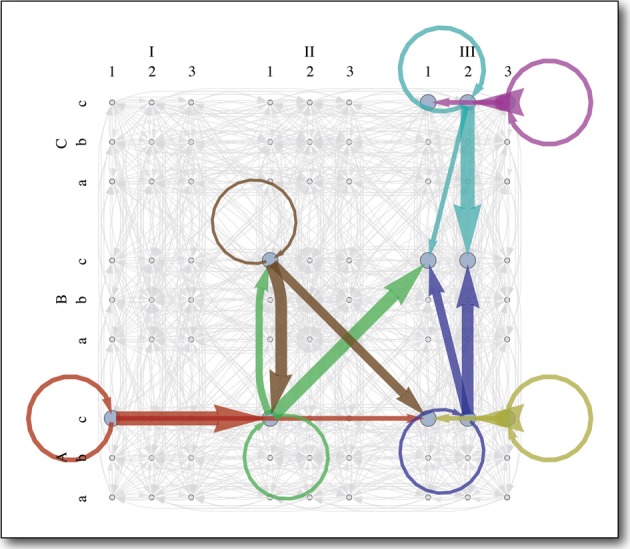
**The learned single-arm MDP planner**. The 4D state space is labeled as follows: shoulder flexion/extension (1,2,3), arm abduction/adduction (a,b,c), lateral/medial arm rotation (I,II,III), elbow flexion/extension (A,B,C). Each color represents an *interesting* state-action, which often takes the agent to some unexpected state. Each arrow of a particular color represents a state transition probability and the weight of the arrow is proportional to the magnitude of that probability. Arrows in gray represent *boring* state-actions. These work as expected, reliably taking the agent to the intended goal state, to which they point.

The learned MDP is pictured in Figure 7. Since the workspace of the arm is unobstructed, most of the state-actions behave as expected, reliably taking the agent to the intended goal state (Equation 3). These deterministic state-actions, shown in gray, are *boring*. The *interesting* ones, each shown in a different color, took the agent to a novel state, which was not represented in the initial state transition distribution for that state-action. Since the environment is static, one would expect even these novel state transitions to be deterministic (Equation 4), and some of them are (red, yellow, purple, light blue). However, the other state-actions (green, brown, and dark blue) sometimes lead to the intended goal state and sometimes lead to one other state, despite the static constraints and the fact that each state-action always runs the same control code.

The fact that static constraints do not necessarily lead to deterministic state transitions is quite interesting. It shows that the iCub, an advanced, light-weight, cable-driven robot, exhibits important non-linearities, due to its mechanics and/or embedded control systems, which prevent it from reliably and repeatably executing precise motions. Therefore, a *plan first, act later* approach, such as PRM planning, will never work well on robots such as the iCub. Plans will sometimes fail at runtime, and not necessarily in a repeatable manner. In fact the lighter and more flexible robots get, the more non-linearities will dominate their dynamics, which is an important motivation for continuing to develop more robust solutions, such as the MDP motion planning presented here.

### 3.2. Discovering the table with a multi-agent RL system

In the second experiment, we control both of the iCub's arms and its torso, 12 DOF in total. A hypercube in 12 dimensions has 4096 vertices, and a rank 3 hyper-lattice has 531,441 vertices. Clearly, uniform sampling in 12 dimensions will not yield a feasible RL problem. Therefore, we have parallelized the problem, employing three curious agents that control each arm and the torso separately, not having access to one another's state. The state-action spaces for the arms are exactly as described in the previous experiment, and the state-action space for the 3D torso is defined in an analogous manner (25%, 50%, and 75% of each joint's range of motion), resulting in a 3D lattice with 27 vertices, which are connected to their 2^3^ = 8 nearest neighbors as per section 2.2.1, yielding 27 × 8 = 216 state-actions.

**Figure 8:**
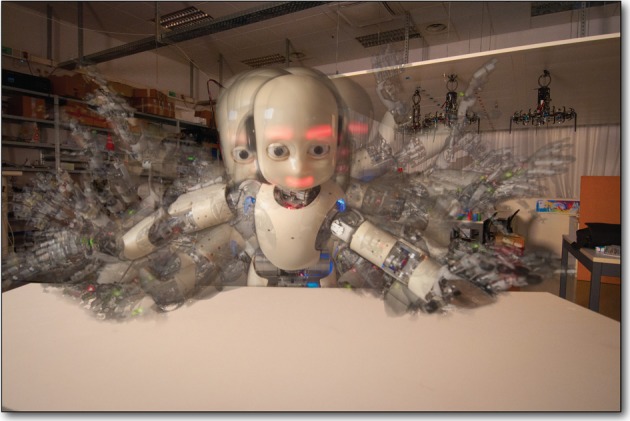
**Autonomous exploration**. This composite consists of images taken every 30 s or so over the first hour of the experiment described in section 3.2.1. Although learning has just begun, we already begin to see that the cloud of robot poses is densest (most opaque) near the table. Note that the compositing technique as well as the wide angle lens used here create the illusion that the hands and arms are farther from the table than they really are. In fact, the low arm poses put the hand or the elbow within 2 cm of the table, as shown in Figure 8.

We place the iCub in front of a work table, and all three learners begin exploring (Figure 8). The three agents operate strictly in parallel, having no access to any state information from the others, however, they *are* loosely coupled through their effects on the robot. For example, the operational space position of the hand (and therefore whether or not it is colliding with the table) depends not only on the positions of the joints in the arm, but also on the positions of the joints in the torso. Thus, we have three interacting POMDPs, each of which has access to a different piece of the complete robot state, and the most *interesting* parts of the state-action spaces are where the state of one POMDP affects some state transition(s) of another.

**Figure 9:**
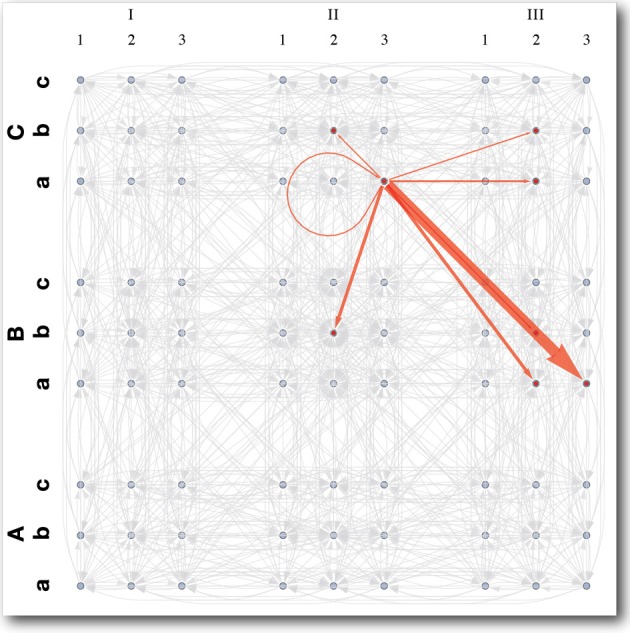
**State space and transition distribution for an *interesting* arm action in multi-agent system**. The 4D state space is labeled as follows: shoulder flexion/extension (1,2,3), arm abduction/adduction (a,b,c), lateral/medial arm rotation (I,II,III), elbow flexion/extension (A,B,C). The red arrows show the distribution of next states resultant of an *interesting* state-action, which causes the hand to interact with the table. Each arrow represents a state transition probability and the weight of the arrow is proportional to the magnitude of that probability. Arrows in gray represent *boring* state-actions. These work as expected, reliably taking the agent to the intended goal state, to which they point.

When the torso is upright, each arm can reach all of the states in its state space, but when the iCub is bent over at the waist, the shoulders are much closer to the table, and some of the arms' state-actions become infeasible, because the robot's hands hit the table. Such interactions between the learners produce state-transition distributions, like the one shown in Figure 9, which are much richer than those from the previous experiment. Moreover these state-actions are the most interesting because they generate the most slowly decaying intrinsic reward of the type shown in Table 4. The result is that the arms learn to avoid constraints as in the first experiment, but over time, another behavior emerges. The iCub becomes interested in the table, and begins to touch it frequently. Throughout the learning process, it spends periods of time exploring, investigating its static arm constraints, and touching the table, in a cyclic manner, as all the intrinsic rewards decay over time in a manner similar to Figure 6.

**Figure 10:**
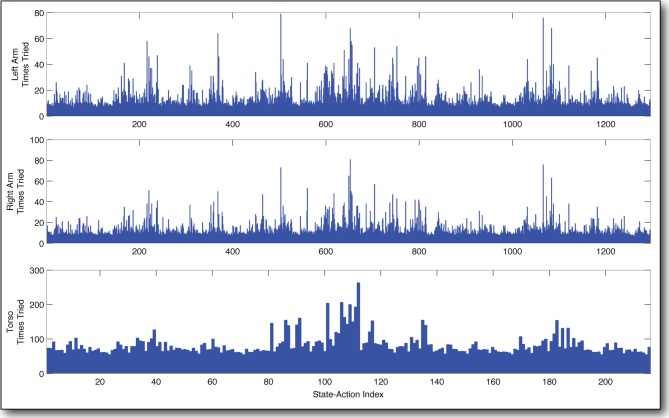
**Frequency of actions taken by three curious agents in parallel**. The most *interesting* actions are selected much more often than the others. They correspond to moving the arm down and leaning the torso forward. This results in the iCub robot being *interested* in the table surface. Note the similarity in the behavior of the two arms.

In Figure 10, we have tabulated the distribution of tries over the state-action space for each of the three learners after 18,000 state transitions, or a little more than two full days of learning. As in the previous experiment, we see that the curious agent prefers certain state-actions, selecting them often. Observing the behavior of the robot during the learning process, it is clear that these frequently chosen state-actions correspond to putting the arm down low, and leaning forward, which result in the iCub's hand interacting with the table. Furthermore, the distribution of selected state-actions for the right arm and the left arm are very similar indeed. This is to be expected, since the arms are mechanically very similar and their configuration spaces have been discretized the same way. It is an encouraging result, which seems to indicate that the variation in the number of times different state-actions are selected does indeed capture the extent to which those state-actions interfere with (or *are* interfered with by) the other learners.

The emergence of the table exploration behavior is quite promising with respect to the ultimate goal of using MDP based motion planning to control an entire humanoid *intelligently*. We partitioned an intractable configuration space into several loosely coupled RL problems, and with only intrinsic rewards to guide their exploration, the learning modules coordinated their behavior, causing the iCub to explore the surface of the work table in front of it. Although the state spaces were generated using a coarse uniform sampling, and the object being explored was large and quite simple, the experiment nevertheless demonstrates that MDP motion planning with AC can empower a humanoid robot with many DOF to explore its environment in a structured way and build useful, reusable models.

#### 3.2.1. Planning in a dynamic environment

There is an alternative way to view the multi-agent experiment. Because the arm does not have access to the torso's state, the experiment is exactly analogous to one in which the arm is the only learner and the table is a dynamic obstacle, moving about as the arm learns. Even from this alternative viewpoint, it is none the less true that some actions will have different outcomes, depending on the table configuration, and will result in state transition distributions like the one shown in Figure 9. The key thing to observe here is that if we were to exploit the planner by placing an external reward at some goal, removing the intrinsic rewards, and recomputing the value function, then the resulting policy/plan will try to avoid the unpredictable regions of the state-action space, where state transition probabilities are relatively low. In other words, training an MDP planner in an environment with dynamic obstacles, will produce policies that plan around regions where there tend to be obstacles.

## 4. Discussion

In this paper we have developed an embodied, curious agent, and presented the first experiments we are aware of, which exploit AC to learn an MDP based motion planner for a real, physical humanoid robot. We demonstrated the efficacy of the AC concept with a simple learning experiment wherein one learner controls one of the iCub humanoid's arms. The primary result of this first experiment was that the iCub's autonomous exploratory behavior, guided by AC, efficiently generated a continually improving Markov model, which can be (re)used at any time to quickly satisfy path planning queries.

Furthermore, we conducted a second experiment, in which the iCub was situated at a work table while three curious agents controlled the its arms and torso, respectively. Acting in parallel, the three agents had no access to one another's state, however, the interaction between the three learners produced an interesting emergent behavior; guided only by intrinsic rewards, the torso and arm coordinated their movements such that the iCub explored the surface of the table.

### 4.1. Scalability

From the standpoint of scalability, the state spaces used for the arms and torso were more than tractable. In fact the time it took the robot to move from one pose/state to another exceeded the time it took to update the value function by approximately an order of magnitude. From an experimental standpoint, the limiting factor with respect to the size of the state-action space was the time it took to try all the state-actions a few times. In these experiments we connected states to their 2^*n*^ nearest neighbors, where *n* is the dimensionality of the configuration space, and we ran the learning experiments for some 12 and 50 h, respectively.

Increasing the number of states in the MDPs would undoubtedly yield a more powerful planner, but it would also increase the time required to learn the models sufficiently. One way to mitigate this effect would be to reduce the number of connections between states. In fact, our impression from qualitative observation of the learning process is that the connectivity of the state space was denser than necessary. Alternatively, we could of course allow the robot to learn for longer. After all, children require years to learn the kinds of skills we are trying to replicate.

### 4.2. Diversity of actions

In these experiments, the implementation of actions (section 2.1) was designed to facilitate motion planning for the purpose of avoiding non-linear constraints on the robot configuration such as such as unwanted collisions. The actions simply set an attractor in configuration space via the MoBeE framework at the Voronoi center of a region of configuration space, which defines a state. The robot then moved according to the transient response of the dynamical system within MoBeE. The result was that our MDP functioned as a sort of enhanced version of a PRM planner, however, the RL framework presented here is in principal capable of much more.

In addition to the position control presented here, our MoBeE framework supports force control in both joint space and operational space, and as far as our RL implementation is concerned, actions can contain arbitrary control code. Therefore, future curious agents for the iCub will benefit from different action modalities, such as operational space reaches or even learned dynamic motion primitives (Schaal et al., 2005).

### 4.3. Bootstrapping the state space

In our view, the main shortcoming of the work presented here is that we have constructed the state-action spaces by hand. In the future, it would be greatly desirable to automate this process, perhaps in the form of an offline process that can run in the background, searching for sets of interesting poses (Stollenga et al., 2013), and incrementally expanding the state-action space. The only part of this proposition, which is unclear, is how to evaluate the quality of the samples that should potentially define new states.

### 4.4. Hierarchies of agents

The experiment “Discovering the table” is promising with respect to the goal of extending our multi-agent MDP motion planning to hierarchies of agents. The *interesting* (most frequently selected) state-actions, as discovered by the current system, constitute each agent's ability to interact with the others. Therefore they are exactly the actions that should be considered by a parent agent, whose job it would be to coordinate the different body parts. It is our strong suspicion that all state-actions, which are not interesting to the current system, can be compressed as “irrelevant” in the eyes of such a hypothetical parent agent. However, to develop the particulars of the communication up and down the hierarchy remains a difficult challenge, and the topic of ongoing work.

### Conflict of interest statement

The authors declare that the research was conducted in the absence of any commercial or financial relationships that could be construed as a potential conflict of interest.

## Footnotes

^1^Generally, throughout this formalism we use uppercase letters to denote sets and lowercase letters to denote points. However, we have made an exception for the states, *s*_*j*_ ∈ *S*, which themselves comprise sets of robot configurations, *s*_*j*_ ⊂ ℝ^*n*^. Although this is somewhat abusive from a set theoretic standpoint, it allows us to be consistent with the standard RL notation later in the paper.

^2^The distance metric employed is the Euclidean norm, in this case: $\sqrt{\left(q-q_{j})\cdot (q-q_{j}\right)}$.

^3^Again, the distance metric employed is the Euclidean norm, in this case: $\sqrt{\left(q_{g}-q_{i})\cdot (q_{g}-q_{i}\right)}$.

^4^We have intentionally not normalized *P* and *T*, to show how they are generated by counting observations of *q*_∞_ ∈ *s*_*j*_. In order to actually compute *D*_*KL*_(*P*||*T*), *P* and *T* must first be normalized.

^5^If there are multiple least-tried state-actions (for example when none have been tried), a random one from the least tried set is selected.
